# Gut Reaction: Environmental Effects on the Human Microbiota

**DOI:** 10.1289/ehp.117-a198

**Published:** 2009-05

**Authors:** Melissa Lee Phillips

Living with each of us—on our skin, in our mucosa, and in our gastrointestinal (GI) tract—are microorganisms whose numbers dwarf the number of our own cells and genes. Although some of these microbes are pathogens, most are harmless or even beneficial. The body’s assortment of microorganisms, collectively called the microbiota, is similar to an organ in that it performs functions essential for our survival. Some microbes produce vitamins and other essential nutrients. Many metabolize food that we can’t digest on our own. They also break down drugs and toxins, and regulate many aspects of innate and acquired immunity, protecting the host from infections and chronic inflammation, as well as possibly many immune-based disorders. And just as with the heart or the lungs, when an environmental agent alters the function of the microbiota, the result can be disease.

Most environment–microbiota research has focused on the gut, home to some 100 trillion microorganisms—the vast majority of our complement of microbes. Shifts in the microbial species that reside in our intestines have been associated with a long list of pathologies, from allergies and autoimmune diseases to obesity and cancer. Some researchers even suspect that the microbiota may play a role in autism spectrum disorders (ASDs).

Each of us carries thousands of bacterial species in our gut along with a few species of other types of organisms. Although all humans have grossly similar microbiota, no two people have exactly the same composition of bacterial species in their guts—in fact, each individual’s microbial consortium may turn out to be as unique as a fingerprint. Yet a study published 22 January 2009 in *Nature* reported that, although individual bacterial species can differ widely between people, the species tend to encode the same metabolic pathways, says coauthor Ruth Ley, an assistant professor of microbiology at Cornell University. “You see the same gene functions regardless of the suite of bacteria present,” Ley says.

Outside influences such as antibiotic use, diet, and psychological stress have shown strong correlations with what lives inside our bodies, and researchers are just beginning to understand how these environmental factors may affect our health. Recent advances in genomic sequencing technologies have pushed the field forward. Whereas scientists once could study only those microorganisms that are easily cultured in a lab—which precludes most of the anaerobic gut microbes—they can now sequence the entire collection of DNA in a microbial sample and identify the component species. This approach, called metagenomics, has been key to many recent advances in understanding the relationship between our microbiota and our health.

## Our Microbial Partners

The infant GI tract is colonized with microorganisms in a complex process that begins during birth and is thought to depend partly on host genetics and partly on the microbes that happen to be in the child’s environment. Babies delivered via cesarean section show different microbial profiles than those born vaginally—whereas vaginally delivered infants are colonized at first by fecal and vaginal bacteria from the mother, infants born through cesarean section are exposed initially to bacteria originating from the hospital environment and health care workers. Research by Giacomo Biasucci et al. in the September 2008 issue of the *Journal of Nutrition* showed that the gut microbiota after cesarean delivery was characterized by an absence of *Bifidobacteria* species, which are thought to be important to the postnatal development of the immune system, whereas vaginally delivered neonates showed a predominance of these species. In general, cesarean-born children also tend to have delayed access to mother’s milk, which has a potent influence on gut microbiota. [For more information, see “Contaminants in Human Milk: Weighing the Risks against the Benefits of Breast-feeding,” *EHP* 116:A426–A434 (2008).]

Throughout the first year of life, the makeup of babies’ gut microbiota can vary widely and is still based largely on the strains of bacteria in their mothers’ bodies as well as those in the immediate environment. Research published by Chana Palmer et al. in the 26 June 2007 edition of *PLoS Biology* showed that, at 1 year of age, infants started to converge toward a microbiota profile that looked more like the adult GI tract, particularly as they began to eat solid foods. Once fully developed in adulthood, the intestinal microbiota is thought to remain quite stable over months or years.

Much of what we know about the influence of microbes on our biology has come from studying “germ-free” rodents, which are born and raised in sterile environments. Because these animals have no microbiota, researchers can deduce which aspects of mammalian biology and physiology normally rely on these symbionts. They can also then inoculate the germ-free animals with various bacteria to see how colonization affects them (the resulting animal is referred to as “gnotobiotic”). Such studies have shown that the gut microbiota is essential for normal development and function of both the GI tract and the immune system.

“Work over the past decade or two links intestinal microbiota very closely with many parameters of host biology in both health and disease,” says Justin Sonnenburg, an assistant professor of microbiology and immunology at Stanford University. In most cases, however, the microbiota–disease connection remains simply a correlation; it’s not yet clear if microbial shifts actually cause disease or if they are simply a reflection of a diseased state. Moreover, exactly how or why our balance of microorganisms sometimes shifts in an unhealthful direction—a condition known as dysbiosis—is frequently unclear.

According to a report by Mohamed Othman and colleagues in the January 2008 issue of *Current Opinion in Gastroenterology*, many problems related to dysbiosis actually may be manifestations of a condition called small intestinal bacterial overgrowth (SIBO). Most gut microbes are supposed to be in the colon, with relatively few in the small intestine. Over time, with changes in intestinal anatomy, motility, and gastric acid secretion, microbes may migrate from the colon up into the small intestine, resulting in SIBO. SIBO is most common in older people, although prevalence reports vary widely. Symptoms of SIBO include diarrhea, abdominal pain, and weight loss resulting largely from malabsorption of micronutrients.

## The Hygiene Hypothesis

Over the past few decades, the increasing incidence of several atopic and autoimmune diseases in developed countries has led to the so-called hygiene hypothesis. The idea is that developing infants need exposure to plenty of microorganisms—both pathogenic and commensal—in order for their immune systems to develop and function properly. According to the hygiene hypothesis, in countries that have very high levels of hygiene (as reflected by the use of refrigeration, pasteurization, water treatment, and food processing to discourage bacterial growth), children aren’t exposed to enough of these microbes. “We’re walking around with a partially ablated microbiota, essentially,” Sonnenburg says.

Several studies have reported a strong correlation between disrupted microbial composition and allergies and asthma. Infants with atopic eczema had lower microbial diversity in their guts as well as fewer species of *Bifidobacteria*, compared with healthy infants, according to a study by Mei Wang et al. in the January 2008 *Journal of Allergy and Clinical Immunology*. A prospective study by Stijn L. Verhulst and colleagues, published in the November 2008 *Journal of Asthma*, found that increased concentrations of anaerobic bacteria in the feces of 3-week-old infants correlated with increased probability of wheezing in the first year of life—a symptom that can be associated with asthma or other lung problems later in life—although *Clostridium* bacteria seemed to be protective.

Dysbiosis has also been seen in inflammatory bowel disease (IBD), a group of intestinal disorders that includes ulcerative colitis and Crohn disease. Some microbial strains—notably of the *Bacteroides* and *Clostridia* species—can produce enterotoxins or possess protein-degrading properties that enhance mucosal permeability and bacterial uptake. In the January 2004 issue of *Gut,* Cyrus Tamboli and colleagues wrote that these strains can colonize the intestinal mucosa and cross the epithelial barrier, whereupon they interact with resident macrophages and trigger the synthesis of proinflammatory cytokines by infected epithelial cells and macrophages. In other cases, IBD patients seem to have an abnormal proinflammatory immune response to normal commensal bacteria, as reported by H. Matsuda et al. in the January 2000 *Journal of Gastroenterology and Hepatology*.

Pervasive antibiotic use may also be disrupting the microbiota among people living in developed countries. Both human and animal studies have shown that even a one-time antibiotic treatment can lead to decreases in bacteria usually considered beneficial, such as *Bifidobacteria* and *Lactobacilli*, as well as increases in potential pathogens such as *Clostridium difficile* and the yeast *Candida albicans*. In the short term, such shifts in microbiota can cause yeast infections and GI symptoms including bloating, abdominal pain, and diarrhea, but recent work suggests the consequences may be much longer-lasting and more serious.

A study by Les Dethlefsen et al., published 18 November 2008 in *PLoS Biology*, reported that most types of intestinal bacteria returned to their pretreatment levels by 4 weeks after the end of a 5-day course of ciprofloxacin, a widely used broad-spectrum antibiotic. But a few types of bacteria failed to recover even 6 months later. A study in the May 2007 issue of *The ISME* [International Society for Microbial Ecology] *Journal* found that levels of some types of gut bacteria remained disrupted up to 2 years after a 7-day course of clindamycin, the drug of choice when treating *Bacteroides* infections.

It’s possible these long-term shifts don’t have any real health consequences, says Janet Jansson, a senior staff scientist in the Earth Science Division at Lawrence Berkeley National Laboratory and senior author of the *ISME Journal* study. “Maybe you lose some species or strains but get new species or strains that take over their functions,” she says. “We don’t know if it matters or not.”

However, Jansson’s study also revealed a “dramatic and persistent” increase in levels of specific resistance genes in *Bacteroides* after clindamycin use. In the people they examined, resistance genes started out at a negligible level but “went up exponentially and stayed at that level for two years,” Jansson says. “Those genes really persist.” [For more information on antibiotic resistance genes, look for next month’s Focus article.]

Perhaps the classic example of how hygiene has altered the microbiota is that of *Helicobacter pylori*. In the 1980s, researchers first isolated the microbe then known as *Campylobacter pylori*, although it is now believed this microbe has been present in most human guts for millennia. Today, however, increased sanitation, hygiene, and antibiotic use have decimated gut populations of this once-ubiquitous microbe. “In developed countries,” wrote Martin J. Blaser in the October 2006 issue of *EMBO* [European Molecular Biology Organization] *Reports*, “new generations are growing up without our ancient companion, *H. pylori*, to orchestrate their gastric hormones.”

Although *H. pylori* has been shown to contribute to gastric adenocarcinoma and lymphoma, as well as ulcers, recent research has revealed another side of this microbe: In the 3 March 2004 issue of the *Journal of the National Cancer Institue*, Weimin Ye and colleagues confirmed that *H. pylori* was associated with a significantly reduced risk of adenocarcinoma in the lower esophagus. *H. pylori* is now also thought to modulate immunologic, endocrine, and physiologic functions in the stomach. In the January 2007 issue of *Seminars in Radiation Oncology*, Rebecca S. Holmes and Thomas L. Vaughan wrote, “The incidence of [*H. pylori*] infection has been declining in the United States and other developed countries, which may contribute to the increasing incidence of [esophageal adenocarcinoma].”

Because factors besides sanitary conditions can confound epidemiologic studies, researchers have also turned to animal models to look for connections between the microbiota and various diseases. In the case of autoimmune disorders, Li Wen et al. reported in the 23 October 2008 issue of *Nature* that depriving mice of a microbiota led to faster onset and more serious disease in a mouse model of type I diabetes. An earlier report by S. Brugman et al. in the September 2006 issue of *Diabetologia* used a rat model for type I diabetes. The authors observed that rats that eventually developed the disease had a lower amount of *Bacteroides* bacteria than rats that did not develop disease. Moreover, antibiotic treatment decreased the incidence and delayed the onset of diabetes, whereas a combination of antibiotic treatment and a special protective diet prevented disease altogether in this diabetes-prone rat.

## Diet and Obesity

Many of the microbial species in the gut metabolize food and extract calories, and some microbes are more efficient at this than others. Because individuals possess slightly different bacterial consortia, it is likely that some people’s microbes harvest more calories, perhaps making this group more likely to become overweight.

In the 2 August 2005 *Proceedings of the National Academy of Sciences*, Ley and other researchers led by Jeffrey Gordon, director of the Center for Genome Sciences at Washington University, found that the gut microbiota of genetically obese mice contained a high percentage of bacteria from the phylum Firmicutes whereas their lean littermates had more bacteria from the phylum Bacteriodetes. In the 21/28 December 2006 issue of *Nature*, Ley reported the same observation in obese and lean human volunteers. She also found that the micro biota of obese people who lost weight through a low-calorie diet shifted to look more like that of leaner people.

It’s difficult to say which came first in some of these studies—obesity or the altered microbiota. In another study from the same Washington University laboratory, also published in the 21/28 December 2006 issue of *Nature*, germ-free mice were colonized with the microbiota of either obese mice or lean mice. Mice that received the obese microbiota gained a higher percentage of body fat than mice receiving the lean microbiota (47.0% versus 0.86%), suggesting that microbiota shifts may contribute to obesity onset. The researchers also found that the obese microbiota, which contained more genes involved in breaking down sugars, appeared to actually harvest more energy from the same diet than did the lean microbiota. The transplant experiments provided functional evidence that this difference may be biologically relevant.

Along with the observation that different microbes and microbial combinations may influence body weight differently, other effects are being defined with respect to changes in human metabolism. For example, dysbiosis has been identified as inflammatory factors that promote insulin resistance and weight gain, as Yolanda Sanz et al. pointed out in a review published online 3 December 2008 ahead of print in *Interdisciplinary Perspec tives on Infectious Diseases*.

Even when not related to obesity, dietary differences appear to influence our microbiota, although exactly how is still somewhat of a mystery, says Nita Salzman, an assistant professor of pediatrics at the Medical College of Wisconsin. Breastfed infants tend to have more *Bifidobacteria* and *Lactobacilli* in their guts than do formula-fed infants. In a study reported by Johan Dicksved et al. in the April 2007 issue of *Applied and Environmental Microbiology*, children raised according to the “anthroposophic” lifestyle touted by philosopher Rudolf Steiner—with restricted antibiotics and plenty of microbe-rich fermented foods—showed higher microbial diversity than farm children, whose diets included more farm-produced animal products.

“The diet definitely can impact the [microbial] composition,” Ley says. It’s also likely that preservatives and other chemicals in our food, as well as the antibiotics and chemicals that we feed our livestock, influence our microbiota, says Salzman. “All of these things probably have an impact on our colonization,” she says. But what exactly such changes mean for health is still obscure.

## The Brain–Gut Connection

Some research suggests the microbiota may also be implicated in neurologic conditions. “There are multiple interfaces where the microbiota could impact our nervous system,” Sonnenburg says. Enteric neurons innervate the gut and transmit signals from it to the nervous system. The microbiota also produces metabolites that are absorbed into the bloodstream, and some of these metabolites can cross the blood–brain barrier. But other studies have failed to find evidence of any connection between the microbiota and neurologic problems, and details of potential mechanisms are still scarce. “There is a good theoretical basis for the microbiota having an impact on our nervous system and, potentially, on cognition,” Sonnenburg says. “But we’re at the very early stages of [study].”

Psychological stress appears to reduce the numbers of *Lactobacilli* species in the human gut while increasing the growth of pathogens such as *Escherichia coli* and *Pseudomonas* species, as reported by Femke Lutgendorff et al. in the June 2008 issue of *Current Molecular Medicine*. In animal work published by Siobhain M. O’Mahoney and colleagues in the February 2009 issue of *Biological Psychiatry*, rat pups were stressed by separating them from their mothers for 3 hours daily between postnatal days 2–12 (the pups still received mother’s milk). Compared with controls, the stressed pups showed markedly altered fecal microbiota. Similar findings have been reported in infant rhesus monkeys separated from their mothers and in adult rats exposed to chronic psychological stress.

The disease ramifications of stress-induced changes are not yet clear, but the scientific literature contains some intriguing possibilities. Psychological stress has been reported to exacerbate both IBD and another intestinal disorder, irritable bowel syndrome (IBS), which is characterized by a variety of intestinal symptoms such as abdominal pain, bloating, and constipation. Multiple studies—including work by Anna Kassinen et al. in the July 2007 issue of *Gastroenterology* and by Mazen Issa et al. in the October 2008 issue of *Inflammatory Bowel Diseases*—have also identified an abnormal gut microbiota in people with these disorders. Still, as Sunny Singh and colleagues pointed out in a review published 31 March 2009 ahead of print in the *American Journal of Gastroenterology*, studies of the stress/bowel disorder link are limited by the subjective nature of documenting psychological stress.

Other research has suggested that ASDs may be related to an altered microbiota. Helena Parracho and colleagues reported in the October 2005 *Journal of Medical Microbiology* that 91.4% of 58 autistic children studied had a GI disorder, compared with 25% of otherwise-healthy siblings of children with ASDs and none of a group of unrelated healthy children. The fecal biota of children with ASDs consistently contained different *Clostridium* species than that of healthy children, as well as a statistically significant increase in clostridial species overall. The siblings of the children with ASDs exhibited intermediate levels of *Clostridium* species, suggesting that environmental factors and genetics may affect gut populations of these species. The researchers point out that *Clostridium* species produce not only enterotoxins that lead to GI problems but also neurotoxins, which they hypothesize could lead to characteristic signs of ASDs.

In another potential gut–ASD connection, antibiotic use in rats has been shown to alter the animals’ gut microbiota to the point of almost completely inhibiting mercury excretion. Because mercury toxicity is a leading suspect behind ASDs, some researchers hypothesize that high use of antibiotics likewise may inhibit children’s ability to excrete the metal, increasing the risk of these disorders. “Specifically,” wrote James B. Adams and colleagues in volume 70, issue 12 (2007) of the *Journal of Toxicology and Environmental Health, Part A*, “oral antibiotics will reduce the amount of normal gut flora (which demethylate methyl-mercury) and may increase the amount of yeast and *E. coli* (which methylate inorganic mercury), resulting in both higher absorption and decreased excretion of mercury.”

## Finding Treatments

As connections between the microbiota and disease are revealed, many researchers are hopeful that it will be possible to find novel treatments for these conditions by targeting our bacteria. There are burgeoning attempts to treat microbiota-associated illness with probiotics and prebiotics. Probiotic foods and dietary supplements introduce live microorganisms that take up residence in the intestines and are thought to be beneficial for health. They usually contain *Lactobacilli* or *Bifidobacteria* species, although some include nonpathogenic strains of *Streptococci*, *E. coli*, and nematode parasites. Prebiotics are made with ingredients such as inulin, oligosaccharides, lactulose, and resistant starch, which are generally thought to encourage *Lactobacilli* and *Bifidobacteria* growth, although they may stimulate other bacterial species as well, including less desirable *Clostridium* species, as noted by Yanbo Wang in a review published in the January 2009 issue of *Food Research International*.

Among the best-supported evidence for probiotics is for their amelioration of childhood viral diarrheas, Sonnenburg says. “That’s pretty solid and I think widely accepted,” he says. Research is also convincing that, following antibiotic treatment, probiotics can help the microbiota rapidly return to a normal composition along with reducing diarrhea-like bowel movements, as demonstrated by Catherina J.M. Koning et al. in the January 2008 *American Journal of Gastroenterology*.

Many studies have provided evidence for probiotic efficacy in conditions such as allergy development, ulcerative colitis, and Crohn disease. In mice, *Lactobacilli* have shown antidiabetic and antitumor effects. In their *Interdisciplinary Perspectives on Infectious Diseases* review, Sanz and colleagues described preliminary evidence that probiotics and prebiotics with antiinflammatory properties could be effective against obesity, diabetes, and associated disorders. For example, the administration of a probiotic exerted a preventive effect against type 1 diabetes in a nonobese diabetic mice model via immune-modulating mechanisms, as reported in the August 2005 issue of *Diabetologia*.

But research findings regarding probiotic efficacy are not always consistent, Sonnenburg says. “There are some great suggestive studies where we see an impact of probiotics, but there are just as many or more where there’s no impact,” he says.

A review by Darren M. Brenner and colleagues in the April 2009 *American Journal of Gastroenterology* determined that a particular strain of *Bifidobacterium infantis* was the only probiotic studied in 16 randomized controlled trials that consistently showed evidence of improving symptoms of IBS. However, the authors wrote, most of the trials either didn’t use an appropriate study design or didn’t sufficiently report adverse events, so it was impossible to assess the efficacy of other probiotics. Brenner and colleagues wrote that future probiotic studies should use standardized diagnostic criteria to allow appropriate assessment of probiotic efficacy. “We’re very much in the infancy of this field,” says Ley. “There’s so much we don’t understand.”

Still, treating disease with probiotics or prebiotics is one of the most promising frontiers in microbiota research, says Mélanie Gareau, a postdoctoral fellow training at The Hospital for Sick Children in Toronto. Her work, published in the November 2007 issue of *Gut*, has shown that treating stress-induced gut abnormalities with *Lactobacilli* probiotics not only reestablished normal gut function and bacterial behavior but also reduced stress hormone levels. “The role of probiotics in maintaining the colonic microbiota is definitely an interesting aspect of this exciting and rapidly advancing field,” she says.

## Exploring the Metagenome

Other promising aspects of microbiota research include advances in sequencing technologies that allow huge numbers of humans and their microbes to be studied, Ley says. Global surveys of human microbiota, for example, may start to address issues of how geography, history, diet, and culture all feed into determining microbiota structure and function. Genomewide association studies are also coming down the pipeline to examine how genetic variation in the human genome predicts microbiota composition. To study these issues reliably, Ley says researchers need to profile the microbiotas of vast numbers of people.

To provide a foundation to answer some of these questions, the National Institutes of Health launched the Human Microbiome Project at the end of 2007. The project will distribute an estimated $115 million over five years to determine what parts of the microbiota are similar in all humans and what parts differ—and how those differences may relate to disease. The European Commission is funding a related effort, called Meta-genomics of the Human Intestinal Tract, and in October 2008 scientists from around the globe formed the International Human Microbiome Consortium to share data on the human metagenome—comprising the genomes of all our commensal microorganisms—among researchers around the world.

Being able to sequence thousands of microbes quickly and easily will likely open up another field, according to Sonnenburg: the consideration of the individual’s microbiota in the development of personalized medicine. The differences between each person’s microbiota will influence not only their health risks but also how they respond to interventions including probiotic treatment and dietary changes, he says. “We should think about differences in our microbiota being analogous to genetic differences that make our responses distinct,” he says.

Simply knowing the constituents of our microbiota won’t be enough to reach this goal, according to Salzman. Even when we can determine the entire microbiota to the species level and every gene of the meta-genome, “it’s still hard to say which of those things is actually important in driving health or disease,” she says. Efforts to provide such an understanding therefore are now focusing not on the genome sequences of resident bacteria but on the proteins they produce.

In the February 2009 issue of *The ISME Journal*, Jansson’s group released an analysis of the human bacterial proteome—the thousands of bacterial proteins that are expressed in our bodies. “The proteome . . . [tells] you what’s actually going on in the gut,” she says. She and her colleagues found that proteins involved in carbohydrate metabolism and food utilization were very highly expressed, whereas other proteins, such as those involved in motility, were nearly absent. They’re now working on analyzing the microbial proteomes in people with Crohn disease.

Another way of analyzing relevant microbial function is to examine the metabolites produced by the microbes, which reveals not just what the bacteria are expressing but what the body is actually absorbing, Salzman says. A study by William R. Wikoff et al., published in the 10 March 2009 *Proceedings of the National Academy of Sciences*, shows that many aspects of mammalian metabolism rely on the microbiota. In the absence of a microbiota, the metabolites found in the blood of mice were significantly changed, suggesting that an animal’s ability to metabolize drugs—and by extension, many other chemicals—likely relies in part on its community of microbes.

Combining genomic inventories of the microbial species that live inside us with functional analyses of the proteins they express and the metabolites we absorb will “advance this field significantly in terms of understanding what’s going on and how it relates to disease,” Salzman says. “Technological advances have profoundly pushed the field, but I still think that we’re probably at the very beginnings.”

## Figures and Tables

**Figure:**
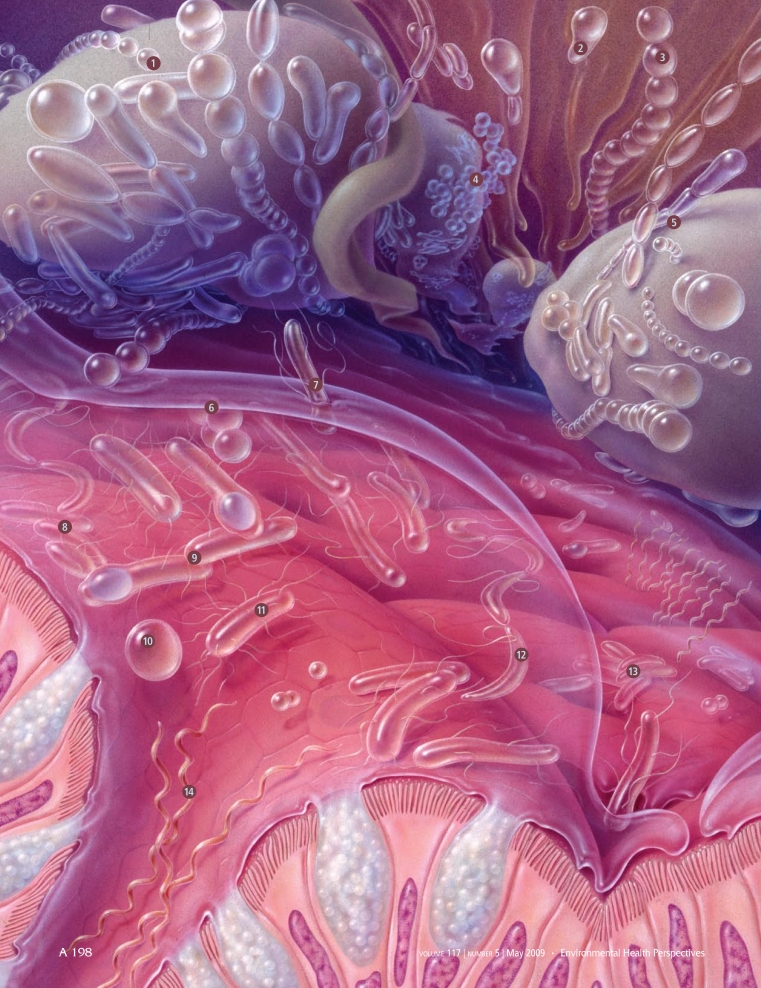
(opposite) The gut contains thousands of microbial species, including: 1) Ruminococcus 2) Bifidobacterium 3) Peptostreptococcus 4) Staphylococcus 5) Lactobacillus 6) Acidaminococcus 7) Fusobacterium 8) Eubacterium 9) Clostridium 10) Coprococcus 11) Escherichia 12) Butyrivibrio 13) Bacteroides 14) Brachyspira

**Figure:**
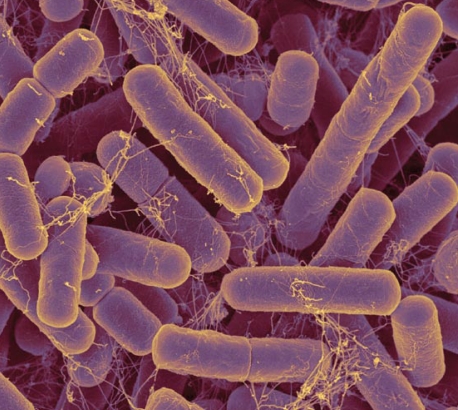
The microbiota is similar to an organ in that it performs functions essential for our survival. And just as with the heart or the lungs, when an environmental agent alters the function of the microbiota, the result can be disease. *Bacteroides* species are some of the most common bacteria in the human gut. They are involved in many important metabolic activities, including fermentation of carbohydrates, utilization of nitrogenous substances, and biotransformation of bile acids and other steroids. But *Bacteroides* can also cause many types of infections and abscesses in the GI tract and elsewhere in the body.

**Figure:**
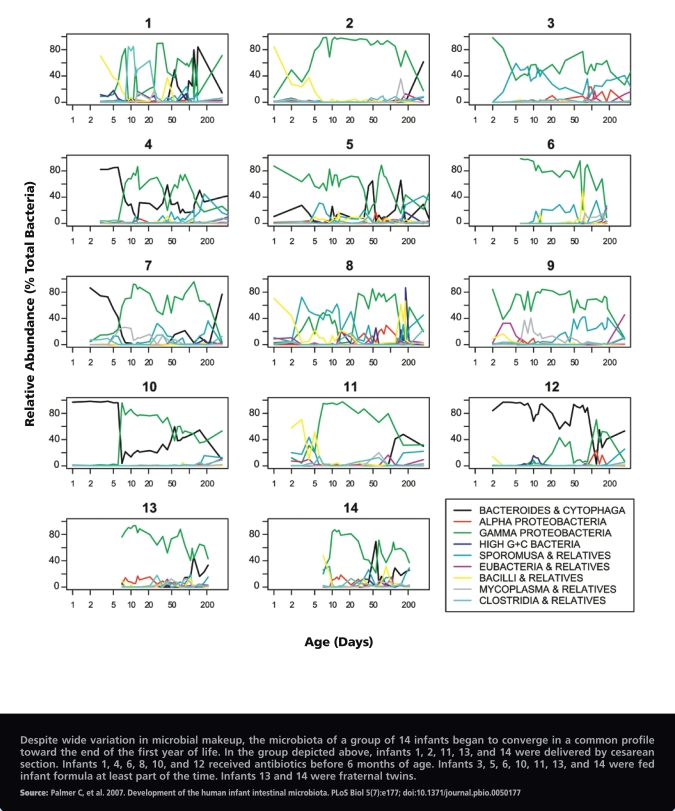
Temporal Profiles of the Most Abundant Gut Microbes as Measured in 14 Infants

**Figure:**
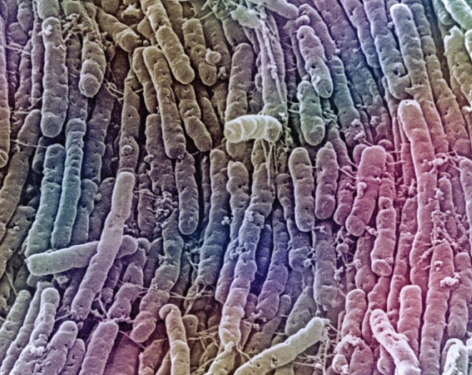
Both human and animal studies have shown that even a one-time antibiotic treatment can lead to long-term shifts in microbial populations. The health consequences of these long-term shifts are still largely unknown. *Clostridium difficile* is often acquired in a hospital setting by patients on antibiotics. Antibiotics alter the normal flora of the intestines, which allows for colonization by *C. difficile*. Once colonized, the bacteria release endotoxins that can cause colitis and severe diarrhea.

**Figure:**
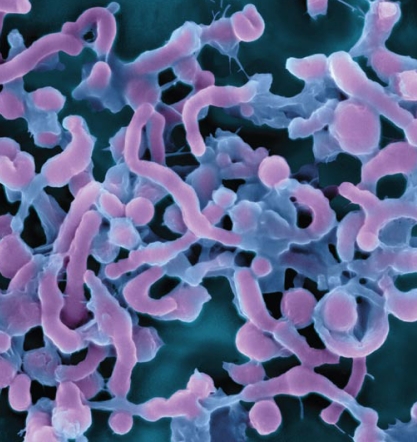
Work over the past decade or two links intestinal microbiota very closely with many parameters of host biology in both health and disease. In most cases, however, the microbiota–disease connection remains simply a correlation; it’s not yet clear if microbial shifts actually cause disease or if they simply reflect a diseased state. *Helicobacter pylori* is the main cause of chronic superficial gastritis and is associated with both gastric and duodenal ulcers, yet it is also associated with a reduced risk of adenocarcinoma in the lower esophagus. It lives in the interface between the surface of gastric epithelial cells.

**Figure:**
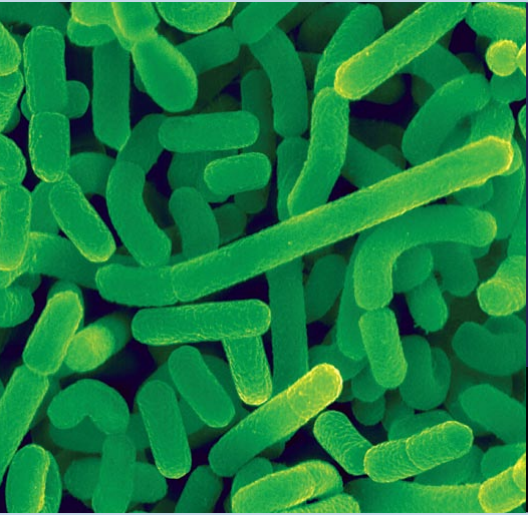
The differences between each person’s microbiota will influence not only their health risks but also how they respond to medical and lifestyle interventions. Being able to sequence thousands of microbes quickly and easily will likely open the door to considering the individual’s microbiota in the development of personalized medicine. *Lactobacillus acidophilus* occurs naturally in the gut, mouth, and vagina. It is also the most commonly used probiotic. This bacterium produces lactase, and *L. acidophilus* supplements are often given to lactose-intolerant individuals.

